# Investigating and Resolving Cardiotoxicity Induced by COVID‐19 Treatments using Human Pluripotent Stem Cell‐Derived Cardiomyocytes and Engineered Heart Tissues

**DOI:** 10.1002/advs.202203388

**Published:** 2022-09-02

**Authors:** He Xu, Ge Liu, Jixing Gong, Ying Zhang, Shanshan Gu, Zhongjun Wan, Pengcheng Yang, Yage Nie, Yinghan Wang, Zhan‐peng Huang, Guanzheng Luo, Zhongyan Chen, Donghui Zhang, Nan Cao

**Affiliations:** ^1^ Center of Translational Medicine The First Affiliated Hospital Zhongshan School of Medicine Sun Yat‐Sen University Guangdong 510080 China; ^2^ NHC Key Laboratory of Assisted Circulation (Sun Yat‐Sen University) Guangzhou 510080 China; ^3^ The Seventh Affiliated Hospital Zhongshan School of Medicine Sun Yat‐Sen University Guangdong 510080 China; ^4^ National & Local Joint Engineering Research Center of High‐throughput Drug Screening Technology State Key Laboratory of Biocatalysis and Enzyme Engineering Hubei Province Key Laboratory of Biotechnology of Chinese Traditional Medicine Hubei University Wuhan 430062 China; ^5^ MOE Key Laboratory of Gene Function and Regulation State Key Laboratory of Biocontrol School of Life Sciences Sun Yat‐Sen University Guangdong 510275 China

**Keywords:** cardiotoxicity, engineered heart tissue, high throughput screening, human pluripotent stem cell‐derived cardiomyocytes, repurposed drugs for COVID‐19

## Abstract

Coronavirus disease 2019 continues to spread worldwide. Given the urgent need for effective treatments, many clinical trials are ongoing through repurposing approved drugs. However, clinical data regarding the cardiotoxicity of these drugs are limited. Human pluripotent stem cell‐derived cardiomyocytes (hCMs) represent a powerful tool for assessing drug‐induced cardiotoxicity. Here, by using hCMs, it is demonstrated that four antiviral drugs, namely, apilimod, remdesivir, ritonavir, and lopinavir, exhibit cardiotoxicity in terms of inducing cell death, sarcomere disarray, and dysregulation of calcium handling and contraction, at clinically relevant concentrations. Human engineered heart tissue (hEHT) model is used to further evaluate the cardiotoxic effects of these drugs and it is found that they weaken hEHT contractile function. RNA‐seq analysis reveals that the expression of genes that regulate cardiomyocyte function, such as sarcomere organization (*TNNT2, MYH6*) and ion homeostasis (*ATP2A2, HCN4*), is significantly altered after drug treatments. Using high‐throughput screening of approved drugs, it is found that ceftiofur hydrochloride, astaxanthin, and quetiapine fumarate can ameliorate the cardiotoxicity of remdesivir, with astaxanthin being the most prominent one. These results warrant caution and careful monitoring when prescribing these therapies in patients and provide drug candidates to limit remdesivir‐induced cardiotoxicity.

## Graphical abstract

By using human pluripotent stem cell‐derived cardiomyocytes and human engineered heart tissue model, this study reveals four potential drugs for COVID‐19, namely, apilimod, remdesivir, ritonavir, and lopinavir exhibiting cardiotoxicity. By using high‐throughput screening, it is found that ceftiofur hydrochloride, astaxanthin, and quetiapine fumarate ameliorate the cardiotoxicity of remdesivir, with astaxanthin being the most prominent one.

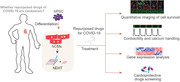

## 1 Introduction

The coronavirus disease 2019 (COVID‐19) pandemic caused by severe acute respiratory syndrome coronavirus 2 (SARS‐CoV‐2) continues to spread worldwide. As of 31 May 2022, the World Health Organization statistics show over 526 million confirmed COVID‐19 cases, with over 6 million deaths. Drug repurposing represents the most promising strategy for the rapid development of therapies of COVID‐19. Many clinical trials have been conducted to investigate the efficacy and safety of several drugs which have been approved or are under investigation for the treatment of other related diseases, such as the anti‐influenza drug favipiravir^[^ ^1^ ^]^ and molnupiravir,^[^ ^2^ ^]^ the anti‐HIV‐drug lopinavir/ritonavir,^[^ ^3^ ^]^ and Janus kinase inhibitors baricitinib^[^ ^4^ ^]^ and ruxolitinib.^[^ ^5^ ^]^

Although the dominant clinical manifestation of COVID‐19 is a respiratory illness, many COVID‐19 patients have underlying cardiovascular disease or have developed acute cardiovascular disorders such as myocardial injury, arrhythmias, acute coronary syndrome, and thromboembolism,^[^ ^6^ ^]^ making COVID‐19 patients more vulnerable to treatment‐induced cardiotoxicity. In addition, drug repurposing may require re‐definition of dose and length of treatment, which may be associated with a new drug safety profile.^[^ ^7^ ^]^ Furthermore, there are several reports of adverse cardiac effects related to some of these potential drugs for COVID‐19.^[^ ^8^ ^]^ The empirical use of lopinavir/ritonavir in COVID‐19 patients was accompanied by an increase in ventricular arrhythmia and QTc prolongation.^[^ ^9^ ^]^ A few clinical trials suggested a potential association between remdesivir and adverse cardiac events.^[^ ^10^ ^]^ Several antivirus drugs that may find uses in COVID‐19 treatments, such as ivermectin^[^ ^11^ ^]^ and oseltamivir,^[^ ^12^ ^]^ are also associated with tachycardia. However, current limited clinical data are far from enough to draw a clear cardiac safety profile of these repurposed drugs in the context of COVID‐19. Hence, understanding the cardiac risk of potential pharmacotherapy being investigated for COVID‐19 is of utmost importance.

Preclinical approaches for evaluating drug‐induced cardiotoxicity via animal models cannot accurately predict human cardiac pathophysiology because of interspecies differences in genetics and cardiac structures.^[^ ^13^ ^]^ Human pluripotent stem cell (hPSC)‐derived cardiomyocytes (hCMs) can spontaneously contract, specifically express proteins found in adult human cardiomyocytes, and can be generated within weeks using defined differentiation protocols. Accumulated evidence has demonstrated that hCMs represent a suitable platform for evaluating drug‐induced cardiotoxicity.^[^ ^13b^ ^]^ Furthermore, human‐engineered heart tissues (hEHTs) constructed using hCMs can better mimic the physiological and anatomical structure of the native heart, not only delivering a means to promote cardiomyocyte maturation but also offering the opportunity to measure contractile function, thus is of great value for cardiovascular disease modeling and drug testing applications.^[^ ^14^ ^]^

Here, we systematically investigated the cardiotoxic effect of a number of potential drugs for COVID‐19 by using hCMs and hCM‐derived hEHTs. We found that apilimod, remdesivir, ritonavir, and lopinavir showed significant cardiac toxicity. By high‐throughput screening of a library containing natural compounds and approved drugs, we identified ceftiofur hydrochloride, astaxanthin, and quetiapine fumarate as protective small molecules that ameliorated the toxicity of remdesivir, with astaxanthin being the most prominent one.

## 2 Results

### 2.1 Assessment of Cardiotoxicity Induced by Repurposed Drugs for COVID‐19 Treatments in hCMs

**Figure 1:**
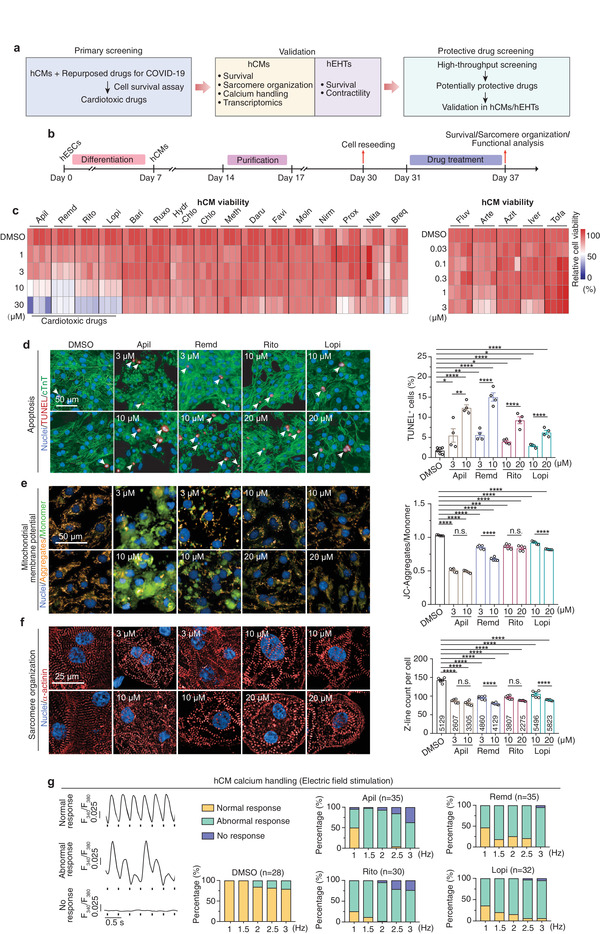
Assessment of cardiotoxicity induced by repurposed drugs for COVID‐19 treatments in hCMs. a) Schematic overview of this study. hCMs, human pluripotent stem cell‐derived cardiomyocytes; hEHTs, human engineered heart tissues. b) Workflow for evaluation of drug cardiotoxicity using hCMs. c) Heatmap showing the cell viability of hCMs treated with DMSO or each drug at the indicated concentrations for 6 days, revealed by calcein‐AM/PI double staining. Drugs tested are as follows: apilimod (Apil), remdesivir (Remd), ritonavir (Rito), lopinavir (Lopi), baricitinib (Bari), ruxolitinib (Ruxo), hydroxychloroquine (Hydro‐Chlo), chloroquine (Chlo), methylprednisolone (Meth), darunavir (Daru), favipiravir (Favi), molnupiravir (Moln), nirmatrelvir (Nirm), proxalutamide (Prox), nitazoxanide (Nita), brequinar (Bre), fluvoxamine (Fluv), artesunate (Arte), azithromycin (Azit), ivermectin (Iver), and tofacitinib (Tofa). *n* = 4 replicates. Nine images were analyzed for each replicate. d–f) Representative (left) and quantitative (right) immunostaining analysis of apoptosis (by TUNEL assay. *n* = 6 replicates for DMSO and *n* = 4 replicates for each drug. Nine images were analyzed for each replicate), mitochondrial membrane potential (by JC‐1 assay, *n* = 5 replicates. 17 images were analyzed for each replicate), and sarcomere organization (*n* = 6 replicates. 24 images were analyzed for each replicate. Number of cells analyzed for each group is labeled within the corresponding bar) in hCMs treated with DMSO or apilimod, remdesivir, ritonavir, and lopinavir at the indicated dose for 6 days. g) Recording of calcium transient response in hCMs with different frequency of electrical field stimulation. Cells were treated with DMSO or each drug at 10 × 10^−6^ m for 6 days. *n* = 28–35 cells for each group, the exact *n* was labeled over the corresponding bar. Data are presented as mean ± SEM. *p ≤ 0.05, **p ≤ 0.01, ***p ≤ 0.001, ****p ≤ 0.0001. n.s., not significant, estimated by one‐way ANOVA with Tukey's post hoc test.

We generated hCMs from the hPSCs with a chemically defined differentiation protocol (Figure S1a, Supporting Information).^[^ ^15^ ^]^ The hCMs expressed typical cardiomyocyte markers, formed well‐organized sarcomere structures surrounded by tons of mitochondria (Figure S1b,c, Supporting Information), showed typical spontaneous calcium transients (Figure S1d, Supporting Information) and responded well to escalated frequency of electrical stimulation (Figure S1e, Supporting Information), and exhibited spontaneous contraction (Video S1, Supporting Information). Upon metabolic purification, the purity of hCMs was up to 98% (Figure S1b, Supporting Information). With these purified hCMs, we then assessed the cardiotoxic effect of a total of 21 drugs (**Figure** **1**a,b). They were either approved or underwent clinical investigation to treat COVID‐19 (**Table** **1**). Cells were treated with these drugs at escalating concentrations for 6 days and the cell viability was assessed by calcein‐AM/propidium iodide (PI) staining analysis. To assess drug cardiotoxicity at clinically relevant concentrations, we tested drug doses range from 0.03 × 10^−6^ to 3 × 10^−6^ m (for drugs whose maximum plasma concentration (*C*_max_) are lower than 1 × 10^−6^ m) or from 1 × 10^−6^ to 30 × 10^−6^ m (for drugs whose *C*_max_ are higher than 1 × 10^−6^ m), respectively. Although hCMs treated with most of the drugs with a concentration up to 30 × 10^−6^ m had no detectable alteration in survival, we observed a dose‐dependent decrease of cell viability in hCMs treated with apilimod, remdesivir, ritonavir, or lopinavir (Figure 1c and Figure S2a, Supporting Information). The four drugs have previously been reported to show antiviral effects against SARS‐CoV‐2 in vitro.^[^ ^16^ ^]^ However, visible cytotoxic effects were exhibited at as low as 1 × 10^−6^ m, a clinically relevant concentration (Table 1). We further observed that apilimod, remdesivir, ritonavir, and lopinavir increased apoptosis of hCMs as determined by the terminal deoxynucleotidyl transferase‐mediated dUTP nick end labeling (TUNEL) assays (Figure 1d). Measurement of mitochondrial membrane potential by JC‐1 staining further confirmed these results (Figure 1e). Moreover, hCMs treated with either of the four drugs showed disorganized sarcomere structures and remarkable decreases in sarcomere number as determined by immunostaining analysis of *α*‐actinin (Figure 1f). Taken together, these data suggest that apilimod, remdesivir, ritonavir, and lopinavir exhibit cardiotoxicity, inducing hCM apoptosis and sarcomeric disarray.

**Table 1:** Information of the repurposed drugs for COVID‐19

	Drug name	Category	Original indication	Cardiotoxic effects	*C*_max_ ^a)^
1	Apilimod	Pikfyve inhibitor	Rheumatoid arthritis, cancer	N/A	0.27 × 10^−6^ m
2	Remdesivir	Adenosine analog, inhibit RNA‐dependent RNA polymerase	Ebola virus	Cardiac arrest,^[^ ^10a,b^ ^]^ heart failures,^[^ ^10c^ ^]^ arrhythmias^[^ ^10c^ ^]^	9.03 × 10^−6^ m
3	Ritonavir	Viral protease inhibitor	HIV‐1 infection	QTc prolongation and arrhythmias^[^ ^9^ ^]^	1.32 × 10^−6^ m
4	Lopinavir	Viral protease inhibitor	HIV‐1 infection	QTc prolongation and arrhythmias^[^ ^9^ ^]^	42.1 × 10^−6^ m
5	Baricitinib	JAK inhibitor	Rheumatoid arthritis	N/A	3.69 × 10^−6^ m
6	Ruxolitinib	JAK inhibitor	Myelofibrosis, polycythemia vera	N/A	7.1 × 10^−6^ m
7	Hydroxychloroquine	Inhibit endosomal maturation by pH elevation	Antimalarial	QTc prolongation^[^ ^41^ ^]^	1.35 × 10^−6^ m
8	Chloroquine	Inhibit endosomal maturation by pH elevation	Antimalarial	QTc prolongation^[^ ^41^ ^]^	1.17 × 10^−6^ m
9	Methylprednisolone	Corticosteroid	Anti‐inflammation, antiallergy	N/A	1.26 × 10^−6^ m
10	Darunavir	Viral protease inhibitor	HIV‐1 infection	N/A	12.7 × 10^−6^ m
11	Favipiravir	Pyrazine analog, inhibit RNA‐dependent RNA polymerase	Influenza	N/A	115 × 10^−6^ m
12	Molnupiravir	Adenosine analog, incorporated into viral RNA, causing RNA nutation	Influenza	N/A	10 × 10^−6^ to 15 × 10^−6^ m
13	Nirmatrelvir	3CL protease inhibitor	N/A	N/A	6.66 × 10^−6^ m
14	Proxalutamide	Androgen receptor antagonist, reduce expression of ACE2	Prostate cancer	N/A	29 × 10^−6^ m
15	Nitazoxanide	Antiprotozoal, inhibit pyruvate‐ferredoxin oxidoreductase	Antiparasitic agents	N/A	17.48 × 10^−6^ m
16	Brequinar	Dihydroorotate dehydrogenase inhibitor	Acute myeloid leukemia	N/A	16 × 10^−6^ m
17	Fluvoxamine	Selective serotonin reuptake inhibitor	Antidepressant	N/A	0.73 × 10^−6^ m
18	Artesunate	Antimalarial	Antimalarial	N/A	0.17 × 10^−6^ m
19	Azithromycin	Macrolide antibiotics	Antibiotic	Ventricular arrhythmias^[^ ^42^ ^]^	0.67 × 10^−6^ m
20	Ivermectin	Macrolide antibiotics	Antibiotic	N/A	0.049 × 10^−6^ m
21	Tofacitinib	JAK inhibitor	Rheumatoid arthritis	N/A	0.135 × 10^−6^ m

^a)^ *C*_max_, maximum plasma concentration. *C*_max_ values were from either U.S. FDA or published literature.

To explore whether the four drugs affect hCM function, we examined the spontaneous intracellular calcium transients in hCMs treated with escalating concentrations of apilimod, remdesivir, ritonavir, and lopinavir for 6 days. The spontaneous calcium transients were monitored by loading cells with the calcium indicator dye Fluo‐4 and examined by confocal laser microscopy. Apilimod increased the time to peak and time to 50% decay of the calcium transients without apparently affecting the calcium transient amplitude at the concentration of 1 × 10^−6^ and 3 × 10^−6^ m (Figure S2c,d, Supporting Information). Notably, no calcium transients were observed in hCMs treated by 10 × 10^−6^ or 20 × 10^−6^ m apilimod because of the cease of spontaneous contraction (Figure S2c,d, Supporting Information). Remdesivir, ritonavir, and lopinavir tended to decrease the calcium transient amplitude. In contrast, only remdesivir and high dosage of lopinavir significantly increased the time to peak and time to 50% decay of the calcium transients (Figure S2c,d, Supporting Information).

Cardiomyocytes display mechanical restitution, whereby a period of time is required after each contraction before another contraction can be elicited.^[^ ^17^ ^]^ Consequently, underlying changes in cardiac refractoriness may go unnoticed at slower beating rates, but become identifiable as the beating frequency is increased. To further assess the calcium handling properties, we subjected the four drug‐treated hCMs to a series of escalating frequencies of electrical field stimulation (from 1 to 3 Hz). We found a sharp decrease in the ratio of cells that could adequately keep pace with the escalating frequencies after being treated with either of the four drugs (Figure 1g). Furthermore, even in the cells capable of keeping pace with the stimulation at 1.5 or 3.0 Hz, we found that drug treatments significantly prolonged the calcium transient durations (Figure S3, Supporting Information). The disharmonic response of the four drug‐treated cardiomyocytes indicates that the calcium handling machinery cannot take up and release calcium stores in time for higher pulse stimulation frequency. Thus, apilimod, remdesivir, ritonavir, and lopinavir impair the intracellular calcium handling properties of hCMs.

### 2.2 Transcriptional Changes in hCMs Induced by Apilimod, Remdesivir, Ritonavir, and Lopinavir Treatment

**Figure 2:**
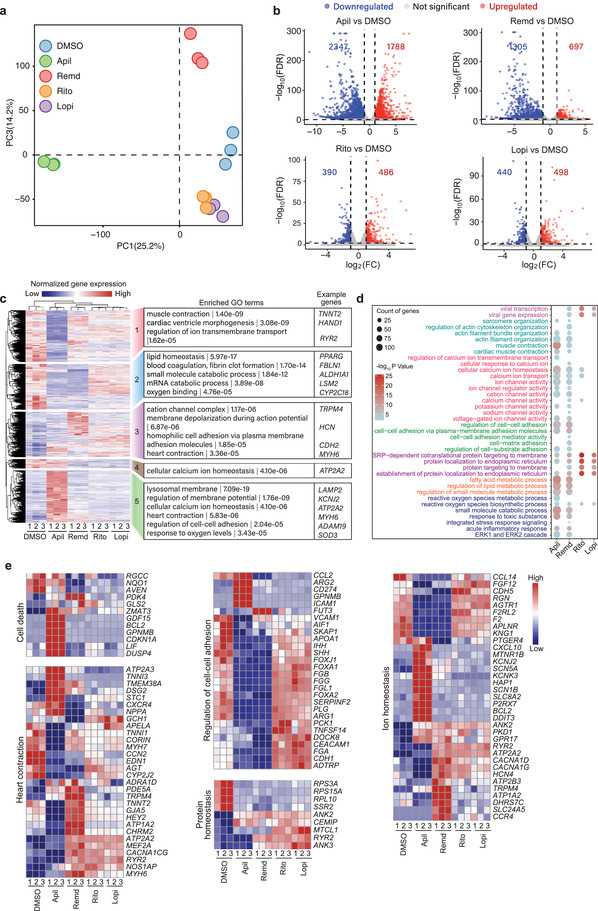
Transcriptional changes in hCMs induced by apilimod, remdesivir, ritonavir, and lopinavir treatment. a) Principal component analysis of the global gene expression profile across all samples revealed by RNA‐seq. b) Volcano plot showing differentially expressed genes (FDR < 0.01 and fold change (FC) > 2) in each drug‐treated group compared to the DMSO group. c) Two‐way hierarchical clustering of all 7897 differentially expressed genes (*p*‐value < 0.05 and FC > 2) in each drug‐treated group compared to the DMSO control. All genes are divided into five clusters. Representative gene‐ontology (GO) terms enriched in each cluster are listed along with their *p*‐values and the example genes. d) GO analysis of differentially expressed genes revealed by RNA‐seq after a 4 day treatment of 10 × 10^−6^ m apilimod and 5 day treatment of 10 × 10^−6^ m remdesivir, 20 × 10^−6^ m ritonavir, or 20 × 10^−6^ m lopinavir in hCMs. e) Expression of the relevant marker genes revealed by RNA‐seq.

To identify transcriptomic evidences of drug cardiotoxicity and potential protective targets, we performed RNA‐sequencing analysis in hCMs with or without the four drug treatments. Principal component analysis (PCA) revealed that hCMs treated by each drug had significantly different expression profiles when compared with the untreated control (**Figure** **2**a and Figure S4ca,b, Supporting Information). As expected, ritonavir‐ and lopinavir‐treated cells shared high transcriptome similarity because both are antiretroviral drugs used to treat human immunodeficiency virus (HIV) infection by inhibiting HIV protease.^[^ ^18^ ^]^ Pairwise comparison of apilimod, remdesivir, ritonavir, and lopinavir with dimethyl sulfoxide (DMSO) revealed many differentially expressed genes (Figure 2b). Not surprising, each drug triggered a unique set of transcriptional changes, with 86 and 102 genes commonly up‐ or downregulated by all four drugs, respectively (Figure S4c, Supporting Information). Consistent with the PCA results, majority of the differentially expressed genes were overlapped between lopinavir and ritonavir (Figure S4c, Supporting Information), once again suggesting their transcriptome similarity. Gene ontology analysis showed that the differentially expressed genes induced by apilimod and remdesivir were mainly related to sarcomere organization, ion homeostasis, and response to oxygen and lipid metabolism, all of which were closely associated with cardiac contraction. Consistent with their less severe cardiotoxic effects, ritonavir and lopinavir minimally altered the transcriptome of hCMs compared to apilimod and remdesivir (Figure 2c,d).

Interestingly, as both ritonavir and lopinavir are protease inhibitors, they introduced gene expression responses associated with viral gene transcription and protein synthesis membrane transport, which may affect hCM function (Figure 2c,d). Next, we evaluated a panel of genes involved in cell death, heart contraction, ion homeostasis, protein homeostasis, and cell–cell adhesion. We found that apilimod, remdesivir, ritonavir, and lopinavir did affect some of the genes involved in the above cellular functions (Figure 2e).

### 2.3 Assessment of Cardiotoxicity Induced by Repurposed Drugs for COVID‐19 Treatments in hEHTs

**Figure 3:**
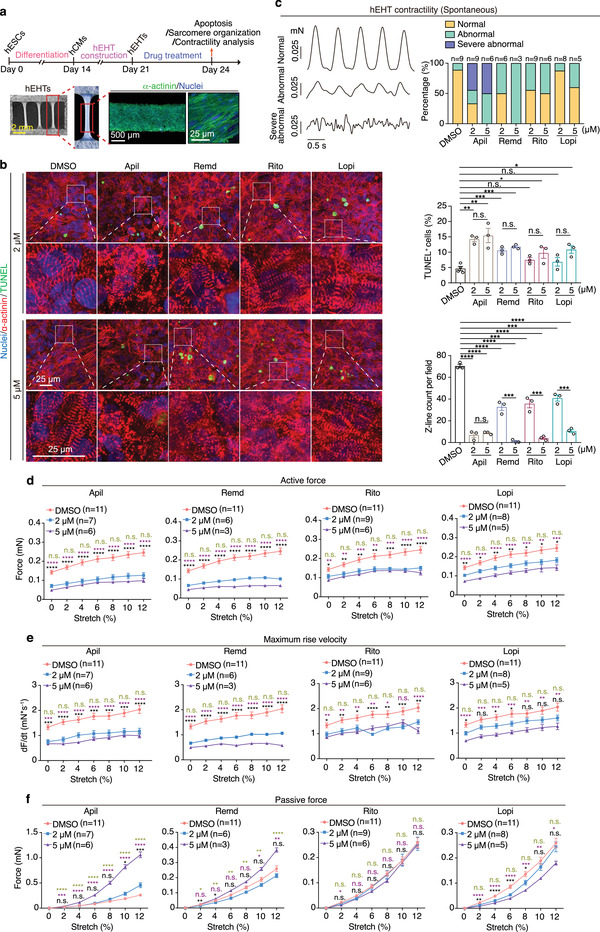
Assessment of cardiotoxicity induced by repurposed drugs for COVID‐19 treatments in hEHTs. a) Schematic of the hEHT‐based assays and the representative hEHT morphology. b) Representative (left) and quantitative (right) TUNEL/*α*‐actinin staining of hEHTs treated with DMSO or each drug at the indicated doses for 3 days. *n* = 3 replicates. Ten images were analyzed for each replicate. c) Active force recording reveals the spontaneous contractility pattern of hEHTs treated with DMSO or each drug at 2 × 10^−6^ or 5 × 10^−6^ m for 3 days. *n* = 3–9 EHTs for each group, the exact *n* is labeled over the corresponding bar. d–f) Parameters of contractile force test during progressive stretch (0%, 2%, 4%, 6%, 8%, 10%, and 12% of tissue length) of hEHTs treated by DMSO or each compound at the indicated concentration for 3 days. *n* = 3–11 hEHTs for each group, 5 traces per hEHT. The exact *n* was labeled within the corresponding graph. These experiments are performed simultaneously and share the same DMSO control. Data are presented as mean ± SEM. *p ≤ 0.05, **p ≤ 0.01, ***p ≤ 0.001, ****p ≤ 0.0001. n.s., not significant, estimated by one‐way ANOVA with Tukey's post hoc test. For (d)–(f), black marks indicate DMSO versus 2 × 10^−6^ m, purple marks indicate DMSO versus 5 × 10^−6^ m, and green marks indicate 2 × 10^−6^ m versus 5 × 10^−6^ m.

To further evaluate the cardiotoxic effects of these drugs at tissue level, we took advantage of the hEHT model (**Figure** **3**a). We treated hEHTs with the four drugs at the clinically relevant concentrations of 2 × 10^−6^ and 5 × 10^−6^ m for 3 days. When compared to the DMSO control group, all drug‐treated groups showed increased apoptosis except for ritonavir and lopinavir at 2 × 10^−6^ m, indicating that apilimod, remdesivir, ritonavir, and lopinavir are toxic to hEHTs (Figure 3b). Once again, hEHTs treated with either of these drugs showed obvious sarcomere disarray accompanied by remarkable decreases in sarcomere number as determined by immunostaining analysis of *α*‐actinin (Figure 3b). To further evaluate the functional effects of these drugs on hEHTs, we tested the spontaneous contractile of hEHTs with or without drug treatments. We found that either of the four drugs led to increase in abnormal beating, which was more severe in the apilimod‐ or remdesivir‐treated hEHTs (Figure 3c). Furthermore, we used a customized contractile force measurement system to analyze the hEHTs’ contraction in stepped raising stretching length (stretching ratio 0%, 2%, 4%, 6%, 8%, 10%, 12%). We found that although the contractile force increased with stretching in all groups tested, it was much lower in the drug‐treated hEHTs, especially in the apilimod‐ and remdesivir‐treated groups (Figure 3d). In addition, maximum rise velocity of the contractile force also dramatically decreased in each of the drug‐treated hEHTs (Figure 3e). Consistently, we observed increases of the passive force with the addition of apilimod or remdesivir, suggesting that they may reduce the toughness of hEHTs, a typical characteristic of healthy tissues^[^ ^19^ ^]^ (Figure 3f). In aggregate, apilimod, remdesivir, ritonavir, and lopinavir are cardiotoxic at the tissue level and weaken hEHT function.

### 2.4 Identification of FDA‐Approved Drugs that Alleviate Remdesivir‐Induced Cardiotoxicity

Remdesivir is the first authorized antiviral drug to treat COVID‐19 under an Emergency Use Authorization.^[^ ^20^ ^]^ Ritonavir and lopinavir have relative low toxicity. Apilimod has been shown to prevent virus invasion by blocking proteases that are crucial for antigen presentation leading to T cell activation.^[^ ^21^ ^]^ This raise concerns that apilimod may suppress the immune system in COVID‐19 patients and theoretically limits its application. Thus, we sought to identify small molecules that can alleviate remdesivir‐induced cardiotoxicity.

By RNA‐sequencing analyses, we found that remdesivir affected many genes involved in cell death and heart contraction. Although supporting the cardiotoxic effects of remdesivir, these changes are more likely to be the downstream consequence rather than a cause of drug toxicity, which are less likely to be suitable targets for developing protective drugs. Phenotypic drug discovery (PDD) relies on cell phenotypes (e.g., morphology, functions, and/or biomarkers) to reveal drug candidates that induce changes in pathological hallmarks. One important advantage of PDD is being not restricted by detailed knowledge of the molecular mechanisms underlying a disease. We therefore decided to perform PDD screening to identify potential protective drugs for remdesivir.

**Figure 4:**
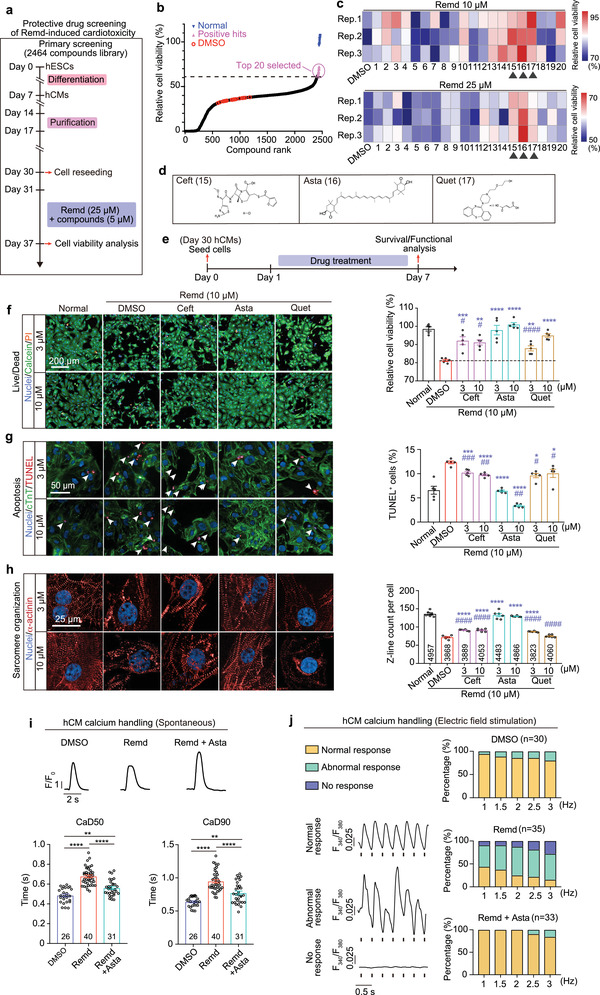
High‐throughput chemical screening identifies FDA‐approved drug candidates that alleviate remdesivir‐induced cardiotoxicity. a–c) The a) workflow and b) primary high‐throughput drug screening results of remdesivir protectors. Top 20 hits were selected for further validation as shown in (c). For validation, cells were treated with 10 × 10^−6^ or 25 × 10^−6^ m remdesivir for 6 days in the presence or absence of the selected screening hits (*n* = 3 replicates). Black triangles indicate the validated chemicals that are protective to both doses of remdesivir‐treatments. d) The chemical structures of ceftiofur hydrochloride (Ceft), astaxanthin (Asta), and quetiapine fumarate (Quet), three validated hits in (b). e) Workflow for evaluation of the protective effects of Ceft, Asta, and Quet. f–h) Representative (left) and quantitative (right) immunostaining analysis of cell viability (by calcein‐AM/PI staining. *n* = 5 replicates. Nine images were analyzed for each replicate), apoptosis (by TUNEL assay. *n* = 5 replicates. 25 images were analyzed for each replicate), and sarcomere organization (*n* = 6 replicates. 24 images were analyzed for each replicate. Number of analyzed cells for each group is labeled within the corresponding bar) in remdesivir‐treated hCMs with or without the co‐administration of Ceft, Asta, or Quet at the indicated dose. i) Representative traces (upper) and averaged parameters (lower) of spontaneous intracellular calcium transient of hCMs treated with DMSO, 10 × 10^−6^ m remdesivir along, or remdesivir plus 10 × 10^−6^ m astaxanthin for 6 days. *n* = 26–44 cells for each group, the exact *n* was labeled within the corresponding bar. CaD, calcium transient duration. j) Recording of calcium transient response in hCMs with different frequency of electrical field stimulation. Cells were treated with DMSO, 10 × 10^−6^ m remdesivir along, or remdesivir plus 10 × 10^−6^ m astaxanthin for 6 days. *n* = 30–35 cells for each group, the exact *n* was labeled over the corresponding bar. Data are presented as mean ± SEM. *p ≤ 0.05, **p ≤ 0.01, ***p ≤ 0.001, ****p ≤ 0.0001. n.s., not significant, estimated by one‐way ANOVA with Tukey's post hoc test. For (f)–(h), * indicates comparing to normal group, # indicates comparing to DMSO control group.

We screened 2464 small molecules from a Bioactive Compound Library comprising natural compounds and US Food and Drug Administration (FDA)‐approved drugs in a high‐throughput PDD assays, by using cell viability as phenotypic readouts (**Figure** **4**a). The top 20 protective hits were selected for further validation (Figure 4b). Three existing drugs, namely, ceftiofur hydrochloride, astaxanthin, and quetiapine fumarate, were confirmed to reduce remdesivir‐induced cardiotoxicity (Figure 4c–f). Ceftiofur hydrochloride is a third‐generation cephalosporin with antibacterial activity.^[^ ^22^ ^]^ Quetiapine is an antipsychotic drug used to treat bipolar disorder and schizophrenia.^[^ ^23^ ^]^ Astaxanthin, an FDA‐approved natural compound, is a xanthophyll carotenoid with various biological activities and beneficial effects on human health, including cardioprotective activity.^[^ ^24^ ^]^ Each of them reduced remdesivir‐induced hCM apoptosis (Figure 4g) and sarcomere disarray (Figure 4h) but have no effects to normal hCMs in the absence of remdesivir (Figure S5, Supporting Information), as revealed by TUNEL and immunostaining analysis, respectively. Since astaxanthin exhibited the most prominent protective effect (Figure 4f–h), we assessed its influence on calcium handling. We found that astaxanthin reversed the remdesivir‐induced alterations in calcium transient duration (Figure 4i). Furthermore, the frequency of abnormal beating caused by remdesivir was relieved dramatically by astaxanthin treatment in the electrical field stimulation analysis, suggesting the functional restore of calcium handling machinery (Figure 4j).

Since astaxanthin is a superior antioxidant, it is reasonable to ask whether it ameliorates the toxicity of remdesivir via scavenging reactive oxygen species (ROS). To address this issue, we measured the level of mitochondrial‐ and cytoplasm‐associated ROS by using the MitoSox red dye or 2′,7′‐dichlorodihydrofluorescein diacetate (DCFH‐DA), respectively. We found that remdesivir treatment did not alter ROS level in hCMs (Figure S6a, Supporting Information). In addition, neither astaxanthin nor four commonly used antioxidants, including *N*‐acetylcysteine, vitamin C, vitamin B1, and reduced L‐Glutathione, could alter the level of ROS in hCMs when co‐stimulated with remdesivir (Figure S6a, Supporting Information). Furthermore, we found that none of the four antioxidants could phenocopy astaxanthin in relieving remdesivir‐induced cell death (Figure S6b, Supporting Information). Taken together, these results demonstrate that the protective effect of astaxanthin on remdesivir‐induced cardiotoxicity is independent of its antioxidant activity.

**Figure 5:**
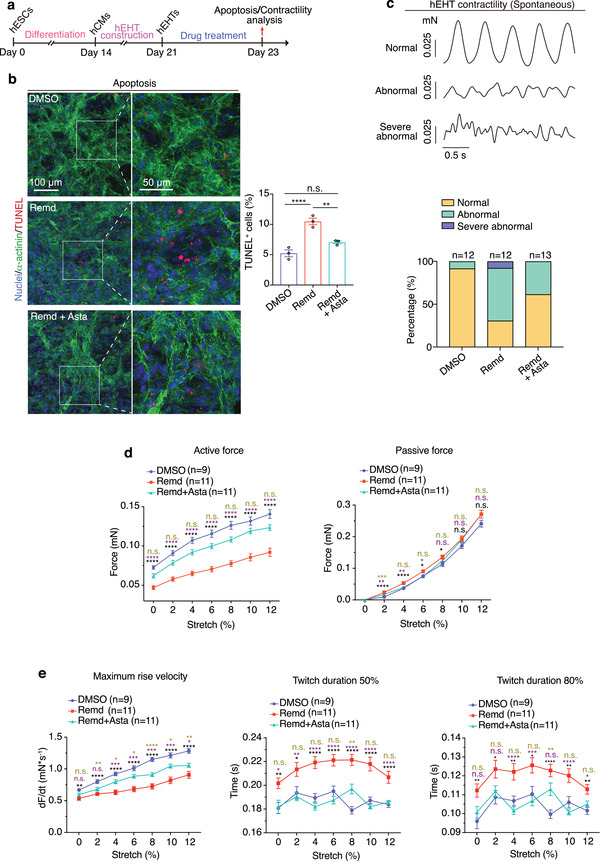
Astaxanthin alleviates remdesivir‐induced cardiotoxicity in hEHTs. a) Schematic of the hEHT‐based assays in evaluating the protective role of astaxanthin. b) Representative and quantitative TUNEL staining of hEHTs treated by DMSO, 2 × 10^−6^ m remdesivir, or 2 × 10^−6^ m remdesivir plus 2 × 10^−6^ m astaxanthin for 2 days. *n* = 3 replicates. Five images were analyzed for each replicate. c) Active force recording reveals the spontaneous contractility pattern of hEHTs subjected to similar treatments in (b) (*n* = 12–13 EHTs for each group, 5 traces per EHT. The exact *n* was labeled over the corresponding bar). d,e) Parameters of contractile force test during progressive stretch (0%, 2%, 4%, 6%, 8%, 10%, and 12% of tissue length) of hEHTs subjected to similar treatments in (b) and (c). *n* = 9–11 hEHTs for each group, 5 traces per hEHT. The exact *n* was labeled within the corresponding graph. Data are presented as mean ± SEM. *p ≤ 0.05, **p ≤ 0.01, ***p ≤ 0.001, ****p ≤ 0.0001. n.s., not significant, estimated by one‐way ANOVA with Tukey's post hoc test. For (d) and (e), black marks indicate DMSO versus Remd, purple marks indicate Remd+Asta versus Remd, green marks indicate DMSO versus Remd+Asta.

Next, we further evaluated whether astaxanthin could resolve the toxicity of remdesivir at the tissue level (**Figure** **5**a). hEHTs were treated with 2 × 10^−6^ m remdesivir and 2 × 10^−6^ m astaxanthin for 2 days. We observed that cell apoptosis was remarkably decreased when hEHTs exposed to remdesivir were concurrently treated with astaxanthin (Figure 5b). Consistently, proportion of abnormal beating (Figure 5c) and decrease in contractile force (Figure 5d,e) induced by remdesivir were also significantly resolved by astaxanthin. Taken together, astaxanthin protects hEHTs against the toxicity of remdesivir.

## 3 Discussion

Numerous drugs have been under investigation to treat COVID‐19, but their potential to cause cardiotoxicity remains unclear. Here, by combining in vitro hCM/hEHT model with the high‐throughput drug screening technology, we provide an alternative platform that can preclinically evaluate the cardiotoxicity of the potential COVID‐19 treatment, investigate the mechanistic toxicology, and identify drug candidates to reduce the cardiotoxicity. Our results demonstrate that apilimod, remdesivir, ritonavir, and lopinavir exhibit cardiotoxicity at clinically relevant concentrations to induce cell death, sarcomere disarray, and dysregulation of calcium handling and contraction. Our findings are in line with the recent reports that remdesivir,^[^ ^25^ ^]^ ritonavir,^[^ ^9^, ^26^ ^]^ and lopinavir^[^ ^9^, ^26^ ^]^ may be associated with adverse cardiac events.

Remdesivir is the first authorized antiviral drug to treat COVID‐19 under an Emergency Use Authorization. We and others^[^ ^25^ ^]^ found that remdesivir induces cardiotoxicity. However, there are no potential drugs available to mitigate remdesivir‐induced cardiotoxicity. Combination of hPSC‐based cell model and PDD has shown tremendous potential in discovering novel leading compounds for treating diseases. For example, by performing a high‐throughput screen of FDA‐approved drugs on hPSC‐derived cell and organoid models, several novel entry inhibitors of SARS‐CoV‐2 have been effectively identified and validated.^[^ ^27^ ^]^ As the safety, pharmacokinetic and manufacturing data are already available, these repurposed approved drugs can be applied quickly to patients. Based on these considerations, we conducted similar high‐throughput PDD screening with a particular focus on FDA‐approved drug library, and identified several potential drugs that alleviate the toxicity of remdesivir, with astaxanthin being the most prominent one.

Astaxanthin is a superior antioxidant.^[^ ^28^ ^]^ Thus, it may likely ameliorate the toxicity of remdesivir via its potent antioxidant activity to scavenge ROS. However, we have shown that remdesivir does not elicit ROS disturbances and other well‐accepted antioxidants do not have similar protective effects as astaxanthin does (Figure S6, Supporting Information). In addition, remdesivir treatment in hCMs did not significantly alter the expression of ROS‐related genes (Figure 2c,d). These findings are in line with the recent report that remdesivir does not alter ROS levels in hCMs.^[^ ^25a^ ^]^ Therefore, astaxanthin seems to protect the hCMs from remdesivir‐induced cytotoxic effects without using its antioxidant activity. There are several reports showing that astaxanthin modulates mitochondrial functions by novel mechanism independent of its antioxidant property. Astaxanthin is able to inhibit the opening of mitochondrial permeability transition pore, slowing down the swelling of mitochondria when added to the isolated mitochondria of rat heart.^[^ ^29^ ^]^ Astaxanthin also has a beneficial effect on mitochondrial quality control through AMPK activation.^[^ ^30^ ^]^ Furthermore, a recent report showed that remdesivir could induce mitochondrial fragmentation and suppress mitochondrial respiration.^[^ ^25a^ ^]^ These studies suggest that astaxanthin may exert the protective effect via acting directly on mitochondrial, which deserves further investigation in the follow‐up studies. Overall, more efforts are needed to understand the underlying mechanisms by which astaxanthin exhibits the protective effect. In addition, further prospective clinical trials are required to assess its efficacy in reducing adverse cardiovascular effects induced by remdesivir in a therapeutic setting.

There are several limitations to this study: 1) number and throughput of the cardiotoxic evaluation should be further increased to meet the continual development of novel COVID‐19 drugs; 2) in addition to cell viability analyses, functional assessments should be included in the future screening of cardiotoxic drugs; 3) transcriptional and epigenetic analyses at the early stage of drug treatment should be performed in follow‐up studies to provide mechanism insights into these COVID‐19 drug‐induced cardiotoxicity; 4) the hEHT model presented here only detects the effects of drugs on cell survival and contractile force, and more functional parameters should be included in the followed works to better distinguish the different functional response to each drug.

In summary, by combining the hPSC‐derived CM model and the tissue engineering technology, we highlight the potential cardiotoxic risk of several repurposed drugs for COVID‐19. More importantly, using remdesivir as an example, our results show that the COVID‐19 drug‐associated cardiotoxicity is preventable and can be ameliorated by high‐throughput screening for small molecule protectors. Also, the function analysis performed in the hEHT platform showed an advantage in increasing drug screening accuracy. These results warrant caution and careful monitoring when prescribing these therapies in patients and provide existing drugs as rapidly translational candidates to limit remdesivir‐induced cardiotoxicity. Finally, our study also highlights the power of human cell/tissue‐based screening platforms for drug testing and discovery.

## 4 Experimental Section

### Cell Culture

H1 human embryonic stem cells (hESCs) (WiCell) were maintained in E8 medium (05990, Stem Cell Technologies) or mTeSR1 medium (85850, Stem Cell Technologies) on Matrigel‐coated dishes (354277, Corning) as described previously.^[^ ^31^ ^]^ Cells were passaged with 0.5 × 10^−3^ m ethylenediaminetetraacetic acid (EDTA)/phosphate‐buffered saline (PBS) when reached 70–80% confluency, with the presence of 5 × 10^−6^ m Rho‐associated protein kinase inhibitor Y27632 (S1049, Selleck) to improve cell viability.

### Differentiation of hESCs into Cardiomyocytes

Cardiomyocyte differentiation was performed as described previously (Figure S1a, Supporting Information).^[^ ^15^ ^]^ The differentiation process began when hESCs reached 70–80% confluence 2–3 days after plating. At day 0, cells were cultivated into differentiation medium 1 supplemented with 6 × 10^−6^ m CHIR99021 (S1263, Selleck). At day 1, cells were cultivated into differentiation medium 2. IWP2 (S7085, Selleck) at 3 × 10^−6^ m was added at day 2 and removed at day 5. hESC‐derived cardiomyocytes (hCMs) were maintained in differentiation medium 1 supplemented with 20 µg mL^−1^ insulin (91077C, Sigma) from day 7 onward. Beating clusters of hCMs were normally observed at day 7. At day 11 to 13, hCMs were metabolically purified using glucose‐ and sodium pyruvate‐free Dulbecco's modified Eagle medium (DMEM, 11966‐025, Gibco) supplemented with 20 × 10^−3^ m lactate (L7022, Sigma) as previously described.^[^ ^32^ ^]^ The formulation of differentiation medium 1 was as followed: DMEM/F12 (C11330500BT, Gibco) supplemented with 10.7 µg mL^−1^ Transferrin (T0065, Sigma), 71 µg mL^−1^ Vitamin C (A8960, Sigma), 14 ng mL^−1^ sodium selenite (S5261, Sigma), and 1× Chemical Defined Lipid Concentrate (11905031, Gibco). The formulation of differentiation medium 2 was as followed: differentiation medium 1 supplemented with 3 µg mL^−1^ heparin (S1346, Selleck). hCMs at 30–35 days of differentiation were used in this study unless otherwise stated.

### Fabrication and Culture of hEHTs

To generate 1.5 × 8 mm 3D human cardiac tissue bundles, polydimethylsiloxane (SYLGARD 184 Dow Corning) molds were designed and microfabricated as previously described.^[^ ^33^ ^]^ Hydrogel solution (24 µL 10 mg mL^−1^ fibrinogen (F3879, Sigma), 12 µL Matrigel (354277, Corning), 24 µL 2x culture medium) was mixed with 1.5 × 10^6^ hCMs in 58 µL culture medium. Following the addition of 2.4 µL 50 U mL^−1^ thrombin (T7201, Sigma), the cell/gel mixture was added to the molds and placed at 37 °C for 15 min to polymerize. Cardiac bundles were removed from the molds and cultured in 12‐well plates on a rocking platform for 7 days before being used in the drug tests as previously described.^[^ ^34^ ^]^ Culture medium was renewed every 2 days.

### Cytotoxicity Assay

hCMs were dissociated with 0.25% trypsin/EDTA (25200072, Gibco) and then seeded into 384‐well plates at the density of 8 × 10^3^ cells per well. 24 h after seeding, cells were exposed to the drugs at the indicated concentrations for 6 days. Culture media were fully changed every 3 days with new drug supplementation. After drug treatment, cells were stained with calcein‐AM/PI/Hoechst for 20 min using calcein‐AM/PI double staining kit (C542, DOJINDO). Images were captured by the Operetta CLS High‐Content Analysis System (PerkinElmer). Number of live cells (calcein‐AM+/PI‐) and dead cells (PI+) was quantified by using the Harmony 4.9 software (PerkinElmer). Number of live cells was used to calculate the “relative cell viability” as follows

(1) $$\begin{array}{c} \mathrm{relarive}\;\mathrm{cell}\;\mathrm{viability}=\frac{\mathrm{number}\;\mathrm{of}\;\mathrm{live}\;\mathrm{cells}\;\mathrm{in}\;\mathrm{each}\;\mathrm{treatment}}{\mathrm{number}\;\mathrm{of}\;\mathrm{live}\;\mathrm{cells}\;\mathrm{in}\;\mathrm{DMSO}\;\mathrm{control}}\times 100\% \end{array}$$

### Immunostaining Assay

Cells and cardiac bundles were fixed in 4% paraformaldehyde (PFA) for 10 min, permeabilized with 0.4%, vol/vol Triton X‐100 for 15 min (for cultured cells) or overnight (for bundles), and blocked with 3% bovine serum albumin for 1 h at room temperature. Then cells were incubated with the primary antibody at 4 °C overnight followed by the fluorescent secondary antibodies for 1 h at room temperature. Hoechst33342 was used to visualize the nuclei. The antibodies used were as follows: *α*‐actinin (A7811, SIGMA), cTNT (MA512960, ThermoFisher), and Alexa Fluor 488‐ or 555‐conjugated secondary antibody (A11001 and A31570, ThermoFisher). Images of hCMs were captured by the Operetta CLS High‐Content Analysis System and Z line number was analyzed by the Harmony 4.9 software. Images of cardiac bundles were captured by Zeiss 710 confocal microscopy and Z line number was analyzed by the Image J software.

### TUNEL Assay

Cell apoptosis was determined using the TUNEL BrightRed Apoptosis Detection Kit (A113‐01, Vazyme Biotech) according to the manufacturer's instructions. Briefly, cells and cardiac bundles were fixed in 4% PFA for 15 min and then permeabilized with 0.4%, vol/vol Triton X‐100 for 5 min at room temperature. Cells and cardiac bundles were then incubated with the TUNEL label reaction solution for 60 min at 37 °C, followed by immunostaining with the cTNT antibody as described above. For hCMs, images were captured by the Operetta CLS High‐Content Analysis System and the number of TUNEL^+^ cells were analyzed and quantified by the Harmony 4.9 software. For cardiac bundles, images were captured by Zeiss 710 confocal microscopy and analyzed by the Image J software.

### Mitochondrial Membrane Potential (MMP) Measurement

MMP was measured using the Mitochondrial Membrane Potential assay kit (C2006, Beyotime Biotechnology) according to the manufacturer's instruction. Briefly, cells were stained with JC‐1 dye for 20 min at 37 °C. Images were captured by the Operetta CLS High‐Content Analysis System. Mean fluorescence intensity of aggregate JC‐1 (red fluorescence) and monomer JC‐1 (green fluorescence) was analyzed by using the Harmony 4.9 software.

### Spontaneous Calcium Transient Measurement in hCMs

hCMs were treated with 1 × 10^−6^ m Fluo‐4 AM (F14201, Thermo Scientific) in the Tyrode's solution (140.0 × 10^−3^ m NaCl, 5.0 × 10^−3^ m KCl, 2 × 10^−3^ m MgCl_2_, 10 × 10^−3^ m HEPES, 1.8 × 10^−3^ m CaCl_2_, 10 × 10^−3^ m glucose, pH 7.4H) for 10–15 minutes at 37 °C. Fluo‐4 AM was then washed off for three times with the Tyrode's solution, and the cells were incubated at room temperature for 15 min before use. Calcium transients were captured in the line‐scan model at the speed of 100 frames s^−1^ using a Zeiss LSM 710 confocal microscope with a 63× objective. During recording, cells were maintained at 37 °C in a heated chamber. The calcium transient data were analyzed with the IDL software (ITT Corporation, White Plains, NY, USA).

### Calcium Measurement in hCMs under Electrical Field Stimulation

Isolated hCMs were seeded on Matrigel‐coated glass coverslip and loaded with 1 × 10^−6^ m Fura‐2 AM (F1221, Invitrogen) in the Tyrode's buffer at 37 °C for 15 min, and then washed with the Tyrode's buffer. Cells were paced with field stimulation (monophasic, 10 V, 5 ms pulses, 1–3 Hz frequency, with 10 s rest between two frequencies) in a perfusion chamber using the IonOptix Calcium and Contractility System (IonOptix). Calcium transients at each frequency were recorded for 5–10 s with a 40× objective. All parameters were calculated offline using the IonWizard 6.3.4 software (IonOptix).

### RNA‐Seq Library Preparation

Total RNA was extracted using the TRIzol reagent (15596026, Invitrogen). Potentially residual DNA was removed by on‐column digestion with RNase‐free DNase (79254, Qiagen). The transcriptome library was generated using the KAPA Hyper Prep Kits (KK8504, Roche). Amplified libraries were sequenced on the Illumina Novaseq platform.

### RNA‐Seq Data Analysis

Paired‐end reads were trimmed with Trimmomatic v.0.36^[^ ^35^ ^]^ with options “ILLUMINACLIP:TruSeq3‐PE.fa:2:30:10:8:true LEADING:10 TRAILING:10 MINLEN:30,” and only those properly paired reads after trimming were retained. The software FastQC v.0.11.5 (https://www.bioinformatics.babraham.ac.uk/projects/fastqc/) was used to assess the data quality of the processed reads. Clean reads were then mapped against the human reference genome (GRCh38) using HISAT2 (v.2.1.0) software^[^ ^36^ ^]^ to generate read alignments for each sample. The quantification of gene expression was performed using the featureCounts v.1.6.0^[^ ^37^ ^]^ with parameters “‐p ‐B ‐T 8 ‐t exon ‐g gene_name.” Differential expression analysis was performed with the R package DESeq2 v.1.30.0.^[^ ^38^ ^]^ Gene ontology enrichment analysis was performed using the R package clusterProfiler v.3.18.0.^[^ ^39^ ^]^ PCA was performed by using “prcomp” function in the default packages of R. Heat maps were generated by the R package pheatmap v.1.0.12 (https://CRAN.R‐project.org/package = pheatmap).

### Assessment of the Contractile Force of hEHTs

Cardiac bundles were treated with DMSO or compound at the indicated concentration for 3 days. Electrically and mechanical stretch stimulated contractile force of cardiac bundles after drug treatment were assessed using a customized force measurement setup as previously described.^[^ ^40^ ^]^ To assess the force–frequency and force–length relationship, cardiac bundles were paced with a 10 V, 2 Hz electrical pulse using a pair of platinum electrodes in Tyrode's solution containing 1.8 × 10^−3^ m Ca^2+^, then sequentially stretched by 2% to 12% of its culture length. Data were analyzed by the MATLAB software (version R2020b (9.9.0.1467703)).

### High‐Throughput Screening of the Protective Compounds

hCMs were seeded into 384‐well plates at a density of 8 × 10^3^ cells per well. 24 h after seeding, the cells were treated with either 25 × 10^−6^ m remdesivir alone or remdesivir with the chemicals from a Bioactive Compound Library (Topscience) consisted of 2464 natural compounds and US FDA‐approved drugs for 6 days. Chemicals at the concentration of 5 × 10^−6^ m were transferred to the plates by using a Tecan Freedom EVO 150 liquid handler.

### Measurement of Mitochondrial and Intracellular ROS

Mitochondrial‐associated ROS and intracellular ROS were measured using MitoSox Red Mitochondrial Superoxide Indicator (M‐36008, ThermoFisher) and DCFH‐DA (S0033S, Beyotime), respectively. For mitochondrial‐associated ROS measurement, hCMs were loaded with 4 × 10^−6^ m MitoSox dye in Hanks' balanced salt solution buffer for 15 min at 37 °C and washed twice with PBS. The MitoSox fluorescence (excitation/emission = 510/580 nm) was detected and quantified by the Operetta CLS High‐Content Analysis System. For intracellular ROS measurement, hCMs were loaded with 10 × 10^−6^ m DCFH‐DA in serum‐free medium for 20 min at 37 °C and washed twice with serum‐free medium. The DCFH fluorescence (excitation/emission = 488/525 nm) was detected and quantified by the Operetta CLS High‐Content Analysis System.

### Statistical Analysis

Data were presented as mean ± SEM from at least three independent biological experiments. Statistical significance was determined using the one‐way analysis of variance (ANOVA) with Tukey's post hoc test for multigroup comparisons. In all cases, *p*‐value ≤ 0.05 was considered statistically significant. Statistical analysis was performed with GraphPad Prism (version 8.0.2).

## Conflict of Interest

The authors declare no conflict of interest.

## Author Contributions

H.X., G.L., J.G., Y.Z., and S.G. contributed equally to this work. N.C., D.Z., and Z.C. conceived the project and wrote the manuscript. H.X., G.L., J.G., and S.G. carried out the experiments. Y.Z. and Y.N. performed bioinformatic analysis. Z.W., P.Y., and Y.W. performed data analysis and assisted with the experiments.

## Supporting information

Supporting Information

Supplemental Video 1

## Data Availability

The data that support the findings of this study are available from the corresponding author upon reasonable request.
