# Supplementary material for: Investigating and Resolving Cardiotoxicity Induced by COVID‐19 Treatments using Human Pluripotent Stem Cell‐Derived Cardiomyocytes and Engineered Heart Tissues
Source: Adv Sci (Weinh). 2022 Sep 2:2203388. Online ahead of print. doi: 10.1002/advs.202203388 (PMC9539280; doi:10.1002/advs.202203388)
Supplement: Supplementary file 1 [file ADVS-9999-0-s002.pdf]

ADVANCED SCIENCE
Open Access

Supporting Information

for *Adv. Sci.*, DOI 10.1002/advs.202203388

Investigating and Resolving Cardiotoxicity Induced by COVID-19 Treatments using Human Pluripotent Stem Cell-Derived Cardiomyocytes and Engineered Heart Tissues

He Xu, Ge Liu, Jixing Gong, Ying Zhang, Shanshan Gu, Zhongjun Wan, Pengcheng Yang, Yage Nie, Yinghan Wang, Zhan-peng Huang, Guanzheng Luo, Zhongyan Chen, Donghui Zhang* and Nan Cao**

Supporting Information

Investigating and Resolving Cardiotoxicity Induced by COVID-19 Treatments Using Human Pluripotent Stem Cell-Derived Cardiomyocytes and Engineered Heart Tissues

He Xu, Ge Liu, Jixing Gong, Ying Zhang, Shanshan Gu, Zhongjun Wan, Pengcheng Yang, Yage Nie, Yinghan Wang, Zhan-peng Huang, Guanzheng Luo, Zhongyan Chen, Donghui Zhang* and Nan Cao**

*Correspondence: chenzhy257@mail.sysu.edu.cn (Z.C.), dongh.zhang@hubu.edu.cn (D.Z.), or caon3@mail.sysu.edu.cn (N.C.)

The supplemental information includes:

Figures S1 to S6

Video S1. spontaneous contraction of day-12 hPSC-derived CMs

Figure S1.

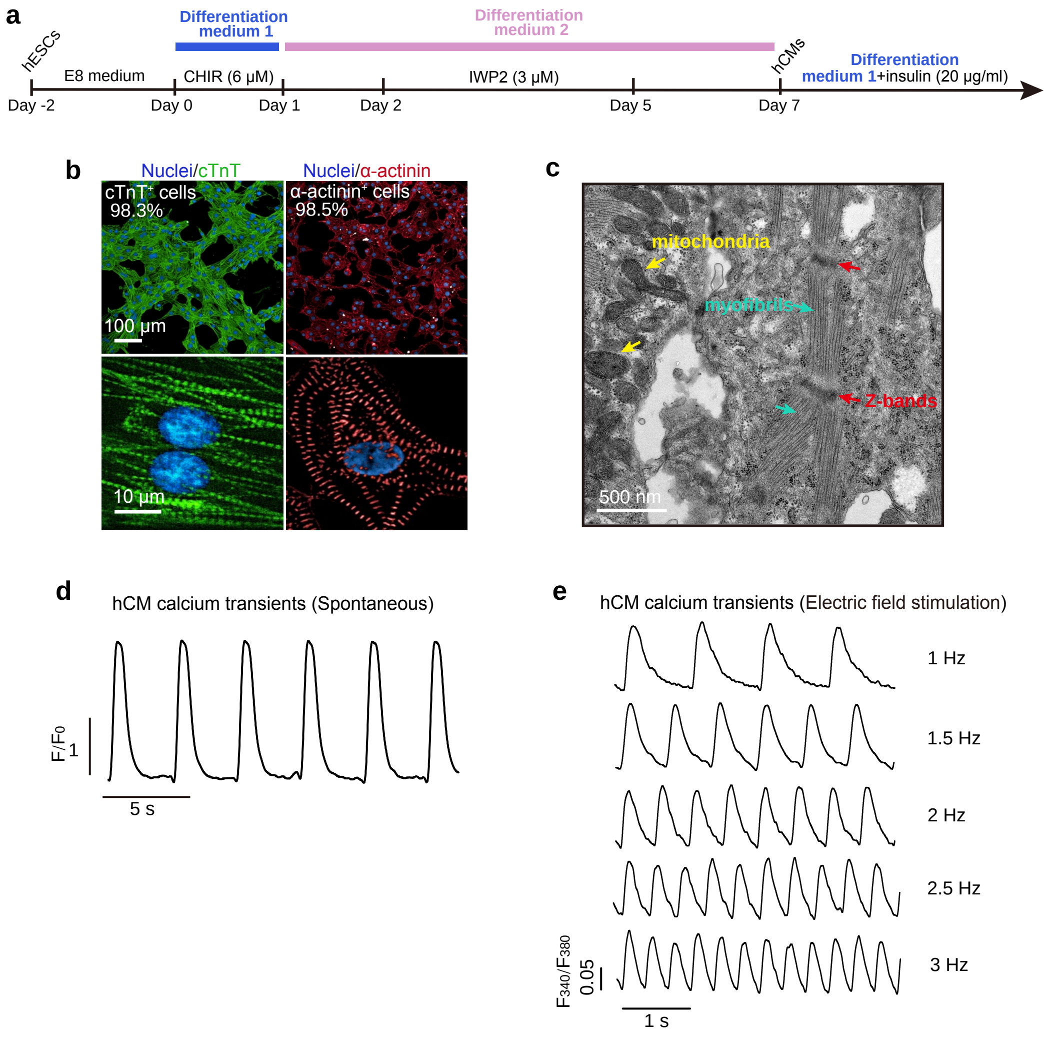

Figure S1. Differentiation and characterization of human pluripotent stem cell derived cardiomyocytes. (a) Schematic of the hCMs differentiation protocol. (b) Immunofluorescence analysis of cardiac markers, including cTnT and α-actinin in cardiomyocytes derived from human pluripotent stem cells at differentiation day 30. Lower panel shows the sarcomere organization of hCMs revealed by images at higher magnification. (c) Transmission electron microscopy image of hCMs showing myofibrils (blue arrows) with Z-bands (red arrows), and mitochondria (yellow arrows). Scale bar, 500 nm. (d) Representative traces of spontaneous intracellular calcium transient in hCMs. (e) Recording of calcium transient response in hCMs with different frequency of electrical field stimulation.

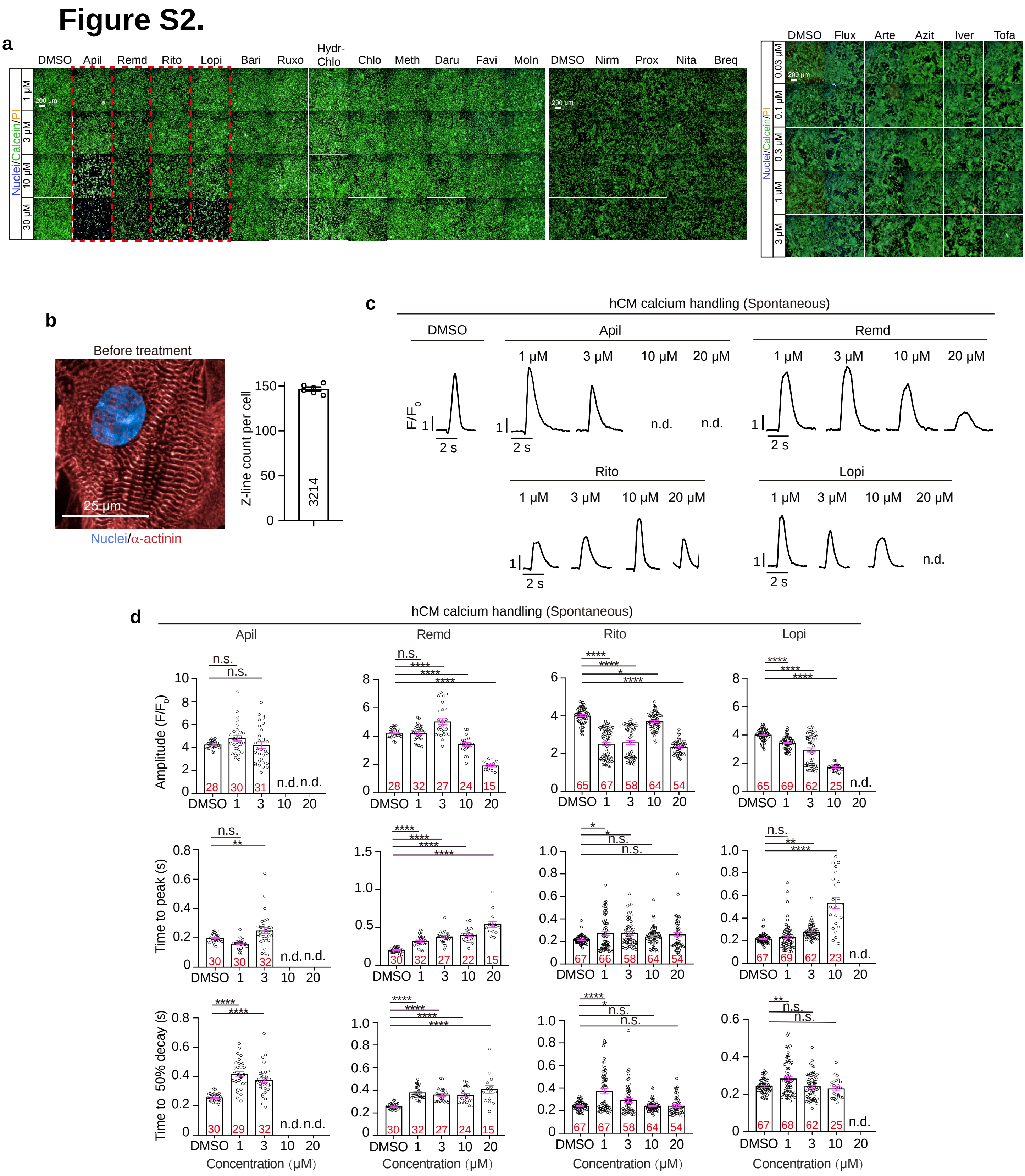

Figure S2. Cellular and functional evaluation of the cardiotoxicity of apilimod, remdesivir, ritonavir, and lopinavir (a) Representative calcein-AM/propidium iodide (PI) double staining of Figure 1b. (b) Representative and quantitative assessment of sarcomere organization of hCMs before drug treatments by immunofluorescence analysis of α-actinin. n = 6 replicates. 24 images were analyzed for each replicate. Number of analyzed cells is labelled within the bar. (c and d) Representative traces (c) and averaged parameters (d) of spontaneous intracellular calcium transient in hCMs treated with DMSO or apilimod, remdesivir, ritonavir, and lopinavir at the indicated dose for 6 days. n = 15-69 cells for each group, the exact n is labelled within the corresponding bar. n.d., not determined. Apil and Remd group, and Rito and Lopi group share the same DMSO control, respectively. Data are means ± SEM. $*P \leq 0.05$, $**P \leq 0.01$, $***P \leq 0.001$, $****P \leq 0.0001$. n.s., not significant, estimated by one-way ANOVA with Tukey's post hoc test.

Figure S3.

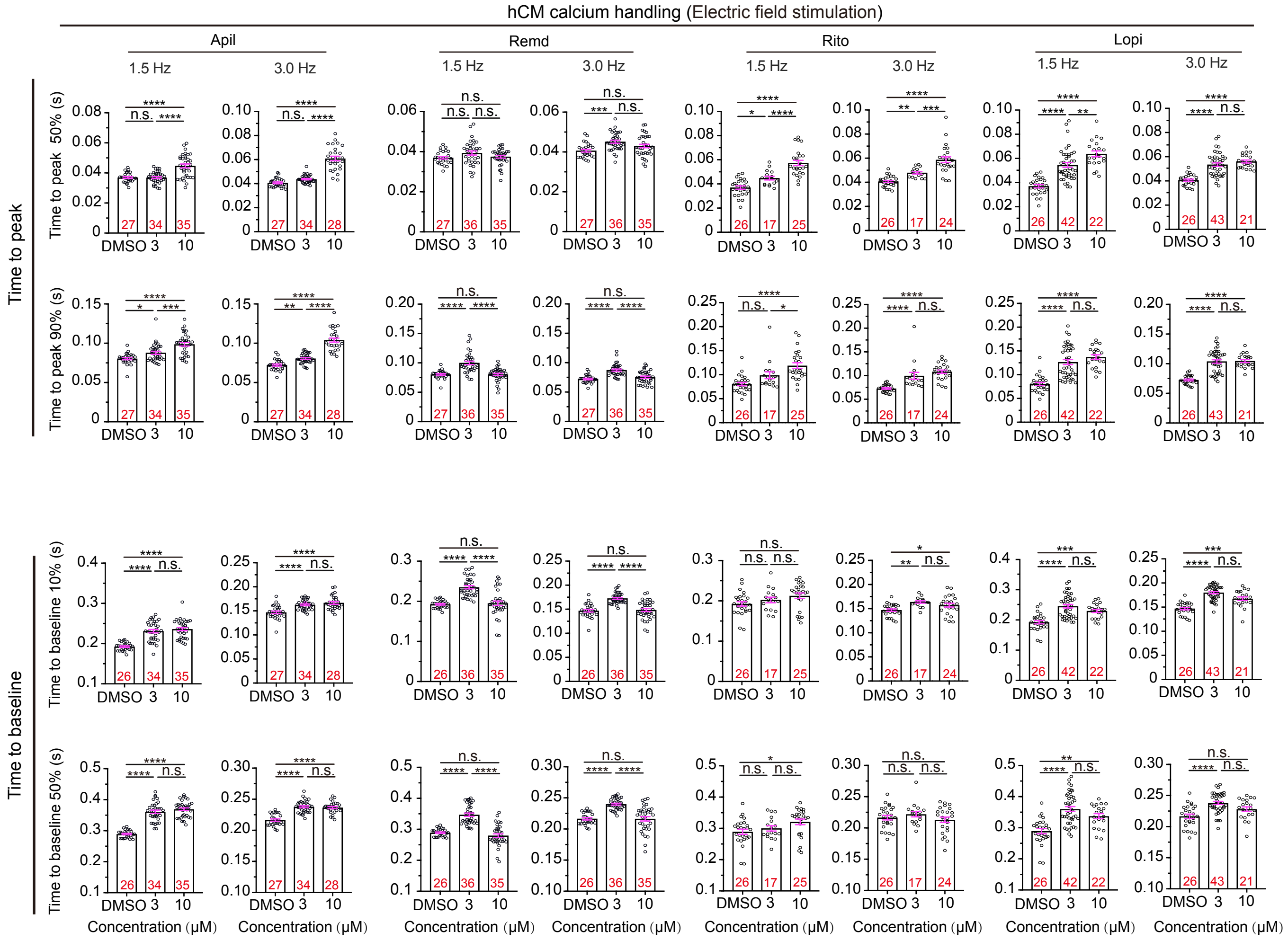

Figure S3. Apilimod, remdesivir, ritonavir, and lopinavir affect hCM function under electrical field stimulation. Averaged parameters of calcium transient of the hCMs treated with DMSO or apilimod, remdesivir, ritonavir, and lopinavir at 3 μM or 10 μM for 6 days with 1.5 Hz or 3.0 Hz electrical field stimulation. n = 17-43 cells for each group, the exact n is labelled within the corresponding bar. Apil and Remd group, and Rito and Lopi group share the same DMSO control, respectively. Data are means ± SEM. *P ≤ 0.05, **P ≤ 0.01, ***P ≤ 0.001, ****P ≤ 0.0001. n.s., not significant, estimated by one-way ANOVA with Tukey's post hoc test.

Figure S4.

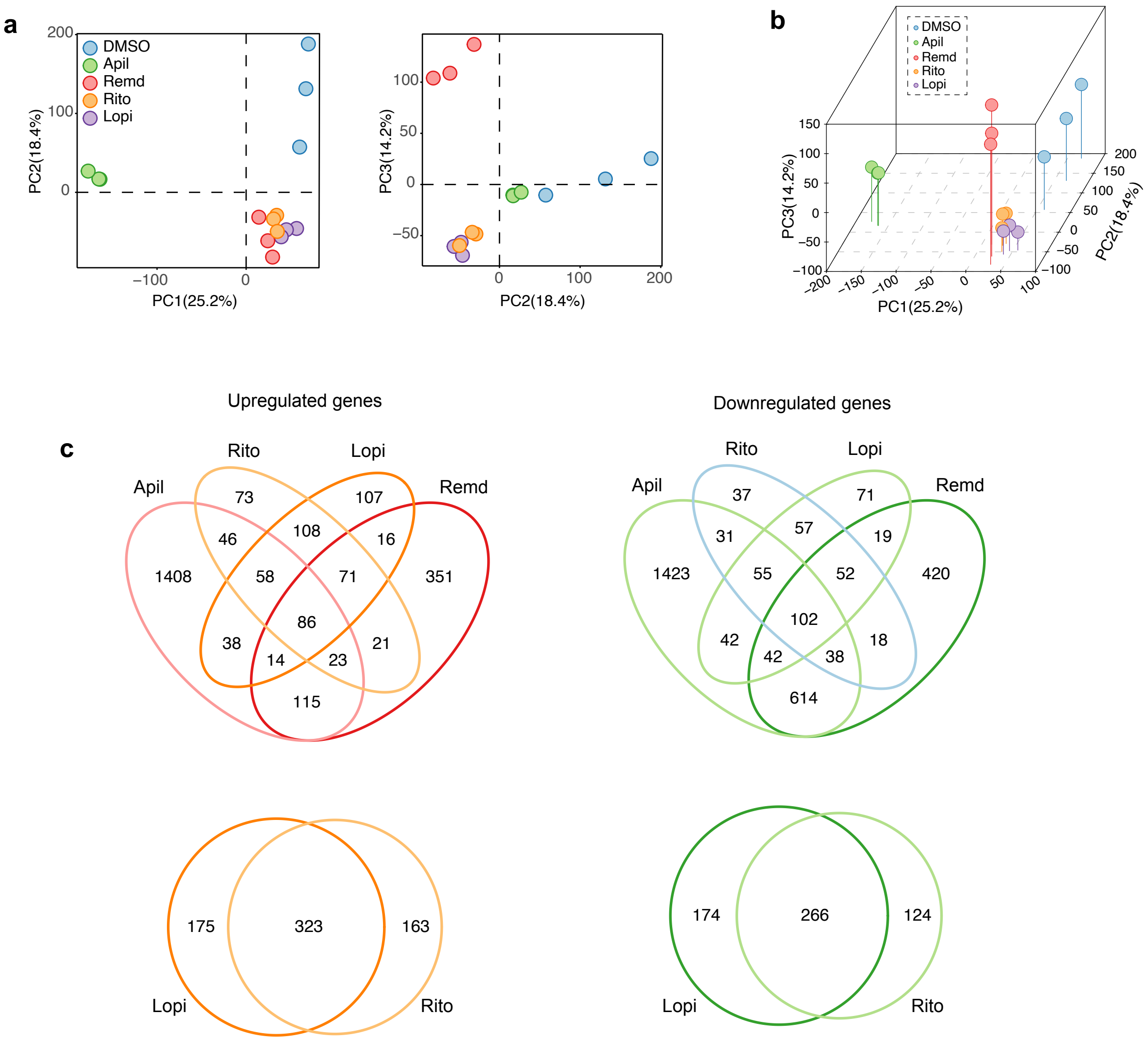

Figure S4. Transcriptional changes in hCMs induced by apilimod, remdesivir, ritonavir, and lopinavir treatment. (a and b) Principal component analysis of the global gene expression profile across all samples revealed by RNA-seq. (c) Venn diagram depicting the overlap of up-regulated (left) or down-regulated (right) genes among the indicated samples.

Figure S5.

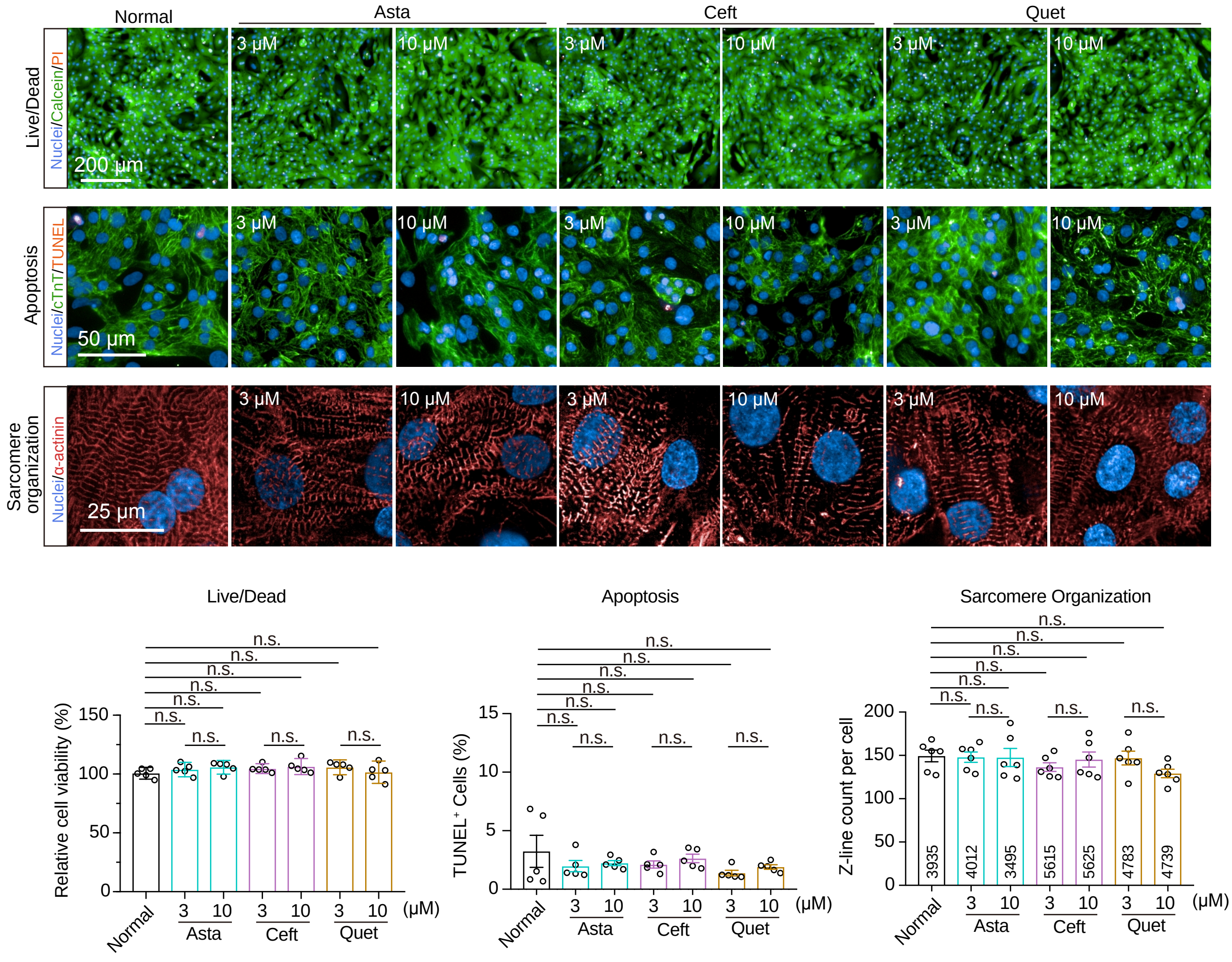

Figure S5. Astaxanthin, ceftiofur hydrochloride, and quetiapine fumarate have little effects on normal uninjured hCMs. Representative and quantitative analysis of cell viability (by calcein-AM/PI staining. N = 5 replicates.9 images were analyzed for each replicate), apoptosis (by TUNEL assay. n = 5 replicates. 25 images were analyzed for each replicate), and sarcomere organization (n = 6 replicates. 24 images were analyzed for each replicate. Number of analyzed cells for each group is labelled within the corresponding bar) in normal hCMs treated with the indicated conditions without co-administration of remdesivir. Data are means ± SEM. n.s., not significant, estimated by one-way ANOVA with Tukey’s post hoc test.

Figure S6.

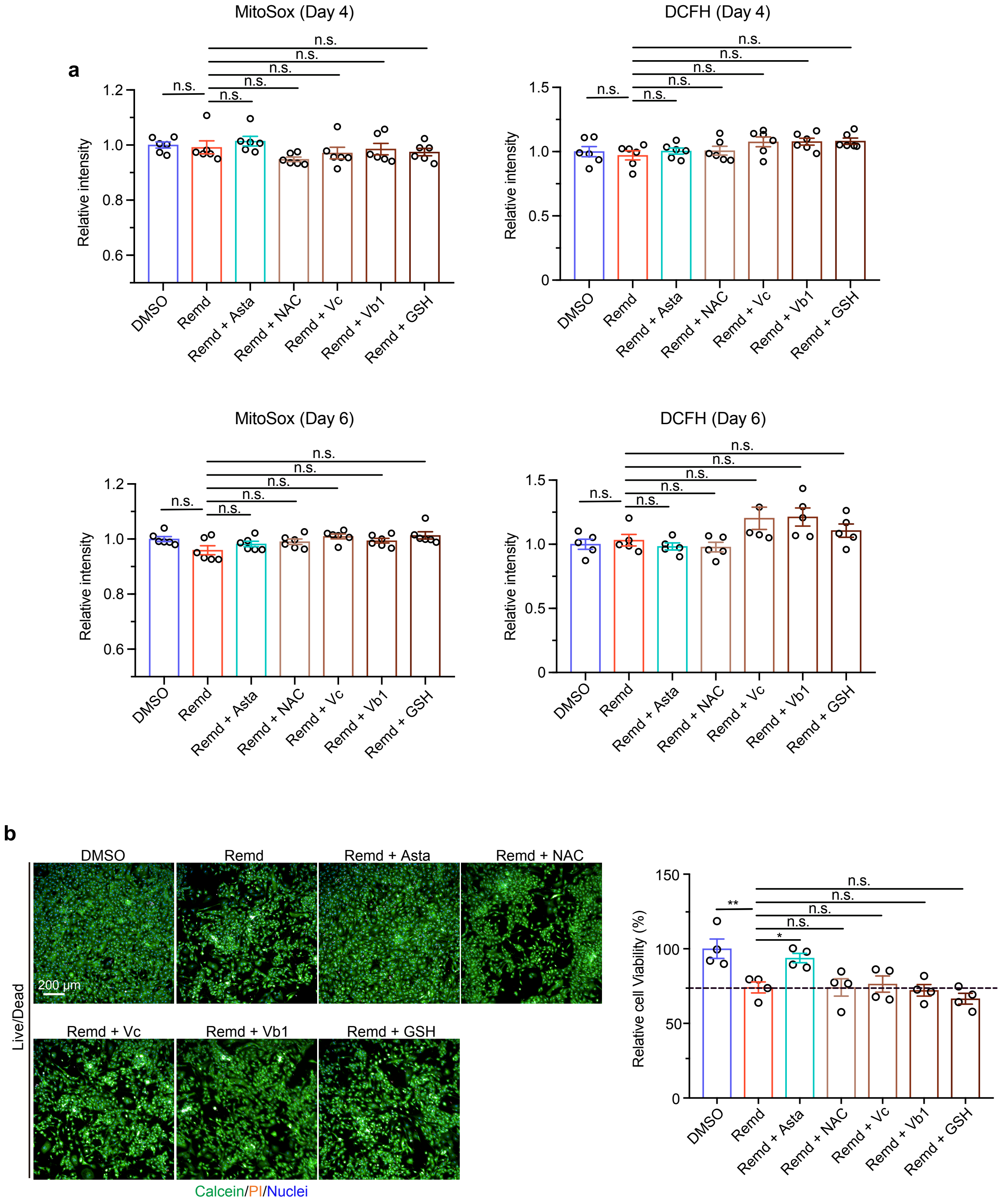

Figure S6. Protective effects of astaxanthin on remdesivir-induced cardiotoxicity is independent of its antioxidant effect.

(a) Measurement of ROS levels by MitoSox or DCFH-DA in hCMs treated with the indicated conditions for 4 or 6 days. Asta, astaxanthin; Vc, vitamin C; Vb1, vitamin B1, NAC, N-acetylcysteine, GSH, reduced L-Glutathione. Drug concentration: remdesivir, 10 µM; Asta, 10 µM; Vc, 50 µg/ml; Vb1,10 µg/ml; Nac, 1mM; GSH , 25 µg/ml. n = 6 replicates. (b) Representative and quantitative staining analysis of cell viability (by calcein-AM/PI staining. n = 4 replicates. 9 images were analyzed for each replicate) in hCMs subjected to similar treatments in a for 6 days. Data are means ± SEM. n.s., not significant, estimated by one-way ANOVA with Tukey's post hoc test.
